# Mouse Mutants of *Gpr37* and *Gpr37l1* Receptor Genes: Disease Modeling Applications

**DOI:** 10.3390/ijms23084288

**Published:** 2022-04-13

**Authors:** Marzia Massimi, Chiara Di Pietro, Gina La Sala, Rafaele Matteoni

**Affiliations:** Institute of Biochemistry and Cell Biology, Italian National Research Council (CNR), I-00015 Monterotondo Scalo, Italy; marzia.massimi@cnr.it (M.M.); chiara.dipietro@cnr.it (C.D.P.); gina.lasala@cnr.it (G.L.S.)

**Keywords:** G protein–coupled receptor, mouse mutant, disease model

## Abstract

The vertebrate G protein–coupled receptor 37 and G protein–coupled receptor 37-like 1 (GPR37 and GPR37L1) proteins have amino acid sequence homology to endothelin and bombesin-specific receptors. The prosaposin glycoprotein, its derived peptides, and analogues have been reported to interact with and activate both putative receptors. The *GPR37* and *GPR37L1* genes are highly expressed in human and rodent brains. *GPR37* transcripts are most abundant in oligodendrocytes and in the neurons of the *substantia nigra* and hippocampus, while the *GPR37L1* gene is markedly expressed in cerebellar Bergmann glia astrocytes. The human GPR37 protein is a substrate of parkin, and its insoluble form accumulates in brain samples from patients of inherited juvenile Parkinson’s disease. Several *Gpr37* and *Gpr37l1* mouse mutant strains have been produced and applied to extensive in vivo and ex vivo analyses of respective receptor functions and involvement in brain and other organ pathologies. The genotypic and phenotypic characteristics of the different mouse strains so far published are reported and discussed, and their current and proposed applications to human disease modeling are highlighted.

## 1. Introduction

The vertebrate *G protein–coupled receptor 37* and *G protein–coupled receptor 37-like 1* (*GPR37* and *GPR37L1*) genes encode seven-transmembrane span proteins with amino acid sequence homology to G protein–coupled receptors for the endothelin and bombesin peptides [1,2,3]. Prosaposin, a secreted cytoprotective glycoprotein, its derived peptides, and synthetic analogues have been reported to interact with and activate both putative receptors [4,5,6].

*GPR37* and *GPR37L1* transcripts are preponderant in several human and rodent brain regions [1]. *GPR37* transcripts are most abundant in oligodendrocytes of fiber tracts and in distinct neuronal populations, such as hippocampal and *substantia nigra* dopaminergic neurons [7]. The *GPR37L1* gene is markedly expressed in cerebellar Bergmann glia astrocytes of newborn, juvenile, and adult mice [8].

The human GPR37 protein has been shown to be a substrate of parkin [7], a proteasomal ubiquitin protein ligase, whose mutant forms cause recessive inherited Parkinson’s disease (PD) [9,10]. Overexpressed GPR37 molecules can become insoluble and accumulate in the endoplasmic reticulum, leading to unfolded protein-induced cell death, whereas parkin-mediated ubiquitination promotes their proteasomal degradation. Furthermore, insoluble GPR37 molecules are found accumulated in brain samples of inherited juvenile PD patients [7].

Several laboratory mouse (*Mus musculus*) strains carrying different targeted mutations or expressed transgenic constructs of the *Gpr37* (Mouse Genome Informatics (MGI) gene ID: MGI: 1313297; formerly termed: *parkin-associated endothelin B receptor-like receptor* or *Pael-R*, *endothelin B receptor-like protein* or *ETBR-LP*) or *Gpr37l* (MGI gene ID: MGI: 1928503; formerly termed: *endothelin B receptor-like protein-2* or *ETBR-LP-2*) gene have been produced and successfully utilized for extensive in vivo and ex vivo analysis of respective receptor’s functions and for investigating their involvement in various brain’s and other organ’s pathologies.

The present review highlights and compares the reported genotypic and phenotypic characteristics of the different mouse strains so far published, with a focus on their current and proposed applications to human disease’s modeling.

A systematic survey and analysis has been conducted of all peer-reviewed publications on *Gpr37* and *Gpr37l1* mouse mutant strains, as included in the following international reference resources: Public Medline (PubMed; National Institutes of Health, https://pubmed.ncbi.nlm.nih.gov/, accessed on 20 March 2022), Europe PubMed Central (EuroPMC; European Bioinformatics Institute, https://europepmc.org/, accessed on 20 March 2022), Mouse Genome Informatics (MGI; The Jackson Laboratory, http://www.informatics.jax.org/, accessed on 20 March 2022), International Mouse Strain Resource Consortium (IMSR; http://www.findmice.org/, accessed on 20 March 2022), Infrafrontier-European Mouse Mutant Archive Research Infrastructure Consortium (Infrafrontier-EMMA; https://www.infrafrontier.eu/, accessed on 20 March 2022), International Mouse Phenotyping Consortium (IMPC; https://www.mousephenotype.org/, accessed on 20 March 2022).

## 2. Mouse *Gpr37* Mutant Strains

The first *Gpr37* constitutive knock-out mutant (KO) strain was produced and functionally studied by Marazziti et al. [11] (MGI allele symbol: *Gpr37<tm2Gtva>*; see Table 1 for details of this and following *Gpr37* and related targeted mutant/transgenic strains), by crossing the corresponding conditional-ready mutant strain, carrying a P1 bacteriophage locus of a crossing over (loxP)-flanked (floxed) *Gpr37*-targeted allele (MGI allele symbol: *Gpr37<tm1Gtva>*), to a transgenic strain which ubiquitously expresses the P1 bacteriophage’s recombination (cre recombinase) protein (Table 1).

The adult homozygous KO mutant mice exhibited reduced striatal dopamine content and increased expression of the dopamine transporter protein at striatal presynaptic plasma membranes, with consequent enhancement of presynaptic dopamine uptake, specific motor deficits, and altered behavioral responses to dopaminergic drugs [11,12]. The homozygous *Gpr37<tm2Gtva>* animals also presented with a striking resistance to 1-methyl-4-phenyl-1,2,3,6-tetrahydropyridine (MPTP), a potent PD-inducing neurotoxin, which causes the specific degeneration of mammalian *substantia nigra* dopaminergic neurons [11].

Further studies showed the constitutive ablation of *Gpr37* to result in decreased expression of endoplasmic reticulum–associated degradation and autophagy pathway markers and impaired phosphorylation of dopaminergic signaling mediators in striatal tissue [13,14], as well as differentiated, gender/age-dependent effects on non-motor behavioral phenotypes [15]. The *Gpr37<tm2Gtva>* KO strain was later used for elucidating the functional expression of the Gpr37 receptor and its proposed ligand, the prosaposin glycoprotein, in the nasal organ’s olfactory ensheathing cells and gonadotropin-releasing hormone neurons [16].

Another *Gpr37* constitutive KO strain was produced and characterized by Imai et al. [18] (MGI allele symbol: *Gpr37<tm1.1Ryot>*), following injection of a cre recombinase-expressing plasmid in oocytes of the corresponding conditional-ready, floxed mutant strain (MGI allele symbol: *Gpr37<tm1.1Ryot>*). The adult homozygous KO mice showed reduced striatal dopamine levels, hypoactivity, altered physiology of striatal neurons, and decreased susceptibility to the MPTP and 6-hydroxydopamine (6-OHDA) dopaminergic neurotoxins [18].

The same authors also produced and studied two transgenic strains, expressing the human GPR37 protein in murine whole brain or midbrain and striatum, respectively (MGI transgene symbols: *Tg(Prp-GPR37)1Ryot* and *Tg(PDGFB-GPR37)20Ryot*). Their phenotypic analysis revealed increased levels of striatal dopamine, hyperactivity, and enhanced susceptibility to 6-OHDA [18].

Each of the two transgenes was also expressed upon crossing to an original *parkin E3 ubiquitin protein ligase* (*Prkn*) constitutive KO strain (MGI allele symbol: *Prkn<tm1Ykt>*), resulting in the manifestation of PD-associated phenotypic traits, with distinctive death of *substantia nigra* dopaminergic neurons, following increased endoplasmic reticulum stress and dopamine toxicity [19].

These findings were consistent with the outcomes of a previous analysis, which had involved the specific, somatic expression of human GPR37 in *substantia nigra pars compacta* neurons, upon striatal injection of recombinant adenoviral vectors in wild-type and *Prkn<tm1Ykt>* null mutant mice [20].

Gpr37’s modulation of endoplasmic reticulum-associated protein degradation was later studied in mouse developing neurons by somatic expression silencing, upon transfection of *Gpr37*-small interfering RNA molecules in neural progenitor cells from C57BL/6N wild-type embryos [41].

A distinct *Gpr37* constitutive KO strain, expressing the bacterial beta-galactosidase (*lacZ*) reporter gene under the control of the endogenous *Gpr37* promoter, was produced by Deltagen Inc. (MGI allele symbol: *Gpr37<tm1Dgen>*), in the context of their high-throughput mouse gene KO production and phenotyping program [21], with initial reporting of gender-specific alteration of body fat/body weight ratio.

The *Gpr37<tm1Dgen>* homozygous strain was applied by several research groups to a variety of studies, which elucidated the expression of the Gpr37 protein in retinal Muller glial cells [22], its functional interaction with hippocampal and striatal adenosine receptors [23,24,25,26,27], and its modulatory role in cortico-striatal synaptic depression [28], as well as in early life stress-induced emotional behaviors [29].

The spatial-temporal modulation of Gpr37 signaling and its behavioral effects were also independently studied by striatum-specific expression of optogenetics-activated algal channelrhodopsin 2-human GPR37 chimeric proteins, upon local injection of recombinant adeno-associated viral (AAV) vectors in adult C57BL/6 wild-type mice [42].

Mice carrying the *Gpr37<tm1Dgen>* KO mutation were used for analyzing Gpr37’s role in brain focal inflammation and progenitor cell dynamics in experimental models of ischemic stroke [30,31].

*Gpr37<tm1Dgen>* null mutants were also instrumental in investigating Gpr37’s neuroprotective action in a specific, dopamine analogue-induced model of PD [32] and further applied to characterize the Gpr37 N-terminal ectodomain as a potential biomarker for PD [33], following its proteolytical cleavage and shedding by a metalloprotease and furin [34].

Other phenotypic analyses of *Gpr37<tm1Dgen>* null mutants investigated the possible activation by the neuroprotectin D1 docosanoid of Gpr37’s signaling pathway in macrophages, and the consequent protection against infection-induced sepsis and inflammation-associated pain [35,36].

*Gpr37<tm1Dgen>* KO mice were also utilized to examine Gpr37’s effects on oligodendrocyte differentiation and myelination [37,38,39].

Qian et al. determined that Gpr37’s attenuating action occurs upon specific stimulation by the bone gamma carboxyglutamate protein (Bglap; synonym: osteocalcin) hormone [39]. They produced and analyzed a novel, oligodendrocyte lineage cell-specific *Gpr37* conditional KO line (MGI allele symbol: *Gpr37<em1Smoc>*). This new genetic tool was obtained after generation of the corresponding conditional-ready, floxed mutant strain (MGI allele symbol: *Gpr37<em2Smoc>*) by clustered regularly, interspaced short palindromic repeats (CRISPR) and CRISPR-associated endonuclease protein (Cas)-mediated gene targeting. The *Gpr37<em2Smoc>* floxed mutant mice were then crossed to mutant animals which express the cre recombinase protein under the control of the endogenous *oligodendrocyte transcription factor 2* (*Olig2*) gene promoter [39].

## 3. Mouse *Gpr37l1* Mutant Strains

A *Gpr37l1* constitutive KO strain was firstly produced and characterized by Min et al. (MGI allele symbol: *Gpr37l1<tm1Mktk>*; see Table 2 for details of this and following *Gpr3l1* and related targeted mutant/transgenic strains) [43].

The same authors also produced and studied a new transgenic strain, specifically expressing the mouse Gpr37l1 protein in cardiomyocytes (MGI transgene symbol: *Tg(Myh6-Gpr37l1)#Mktk*). Comparative phenotypic analyses found significantly higher blood pressure and heart weight/body weight values in homozygous KO mice when compared to transgenic mice, indicating some cardiovascular system-related function of Gpr37l1 [43].

*Gpr37l1<tm1Mktk>*-null mutants were later used to characterize the Gpr37l1 N-terminal ectodomain as a potential cerebrospinal fluid biomarker, upon proteolytical processing by matrix metalloproteases [44].

A distinct *Gpr37l1* constitutive KO strain was produced and functionally studied by Marazziti et al. [8] (MGI allele symbol: *Gpr37l1<tm1.2Gtva>*), upon crossing the corresponding conditional-ready, floxed mutant strain (MGI allele symbol: *Gpr37l1<tm1.1Gtva>*) with a transgenic strain, which ubiquitously expresses the cre recombinase protein (Table 2).

The homozygous null mutant mice manifested specific alterations of postnatal cerebellar development, with premature down-regulation of granule neuron proliferation, precocious Bergmann glia astrocytes and Purkinje neuron maturation and layering, and improved motor abilities and coordination at juvenile and adult age [8].

The detailed comparative analysis of *Gpr37l1<tm1.2Gtva*> homozygous mutant and wild-type littermate control animals also revealed that the Gpr37l1 receptor is specifically expressed in cerebellar Bergmann astrocytes, where it functionally interacts with the patched 1 (Ptch1) protein, the primary cilium’s membrane co-receptor of the sonic hedgehog (Shh) proliferative peptide hormone [8,46]. Moreover, the Gpr37l1 protein was found to specifically interact with cerebellar astrocyte’s megalencephalic leukoencephalopathy-associated membrane proteins [47].

The *Gpr37l1<tm1.2Gtva>* homozygous strain was then crossed to an established *Ptch1* heterozygous KO (MGI allele symbol: *Ptch1<tm1Zim>*) model of cerebellar medulloblastoma (MB). The natural history analysis showed that the genetic ablation of *Gpr37l1* results in the marked delay of post-natal tumor occurrence and the decreased incidence of more aggressive tumor types [48].

Furthermore, the comparative study of cerebellar primary astrocytes from *Gpr37l1<tm1.2Gtva>* homozygous mutant and wild-type littermate pups elucidated the regulatory role exerted by Gpr37l1 and prosaposin ligands on Ptch1 trafficking, Shh production, and Shh-induced astrocyte proliferation [49].

Extensive comparative analyses of the *Gpr37<tm2Gtva>*, *Gpr37<tm1Dgen>,* and *Gpr37l1<tm1.2Gtva>* KO strains had also demonstrated the specific co-expression of the Gpr37 receptor and its proposed prosaposin ligand in developing testis’ Sertoli cells, whose proliferation and maturation are markedly affected in *Gpr37<tm2Gtva>* homozygous null mutant mice [17].

Another *Gpr37l1* constitutive KO strain, expressing the *lacZ* reporter gene under the control of the endogenous *Gpr37l1* promoter (MGI allele symbol: *Gpr37l1<tm1Lex>*), was made publicly available by Lexicon Genetics Inc.’s program for high-throughput mouse gene KO production and phenotyping [50].

The *Gpr37l1<tm1Lex>* and *Gpr37<tm1Dgen>* homozygous strains were used by Giddens et al. to show how the genetic inactivation of each receptor causes an increased susceptibility to epileptic seizures [40]. The same authors also produced and analyzed a novel *Gpr37l1<tm1Lex> Gpr37<tm1Dgen>* double homozygous KO strain, which exhibited a remarkably more severe seizure susceptibility phenotype [40].

The comparative study of the *Gpr37<tm1Dgen>*, *Gpr37l1<tm1Lex>,* and *Gpr37l1<tm1Lex> Gpr37<tm1Dgen>* KO strains had also shown the Gpr37-specific regulation of oligodendrocyte myelin glycoprotein expression and susceptibility to demyelination [38].

Moreover, the quantitative proteomics analysis of brain tissue samples from *Gpr37l1<tm1Lex> Gpr37<tm1Dgen>* double-KO mice revealed an altered pattern of protein expression in comparison with wild-type control samples, including a marked decrease in the expression of the myelin-associated glycoprotein and S100 calcium-binding protein A5 [52].

The *Gpr37l1<tm1Lex>* KO strain was also utilized to characterize the protective effects of Gpr37l1 and proposed prosaposin ligands [56] in brain ischemia and Gpr37l1’s modulation of adult oligodendrocyte generation [51].

Targeted embryo stem (ES) cell clones, carrying a *Gpr37l1* conditional-ready, floxed mutant allele, with a *lacZ* reporter-tagged insertion (MGI allele symbol: *Gpr37l1<tm1a(EUCOMM)Wtsi>*), were made publicly available by the large-scale conditional KO resource of the International Knockout Mouse Consortium (IKMC)—European Conditional Mouse Mutagenesis Program (EUCOMM), for the standardized, high-throughput, genome-wide study of mouse gene function [53].

A *Gpr37l1<tm1a(EUCOMM)Wtsi>* floxed ES cell clone was used by Coleman et al. [54] to obtain the corresponding conditional-ready mouse mutant strain. Appropriate combinations of crossings to two transgenic strains, ubiquitously expressing the *Saccharomyces cerevisiae* flippase (Flp) recombinase or the cre recombinase protein, resulted in the production of heterozygous, reporter-tagged constitutive KO (MGI allele symbol: *Gpr37l1<tm1b(EUCOMM)Wtsi>*) or homozygous, reporter-free constitutive KO (MGI allele symbol: *Gpr37l1<tm1d(EUCOMM)Wtsi>*) progenies [54].

The two strains were then applied for elucidating Gpr37l1’s specific expression and sexually dimorphic modulation of brainstem’s central cardiovascular control and homeostasis [54,55]. Moreover, an experimental reassessment of the cardiovascular phenotypes expressed by the *Gpr37l1<tm1Mktk>*, *Tg(Myh6-Gpr37l1)#Mktk* [43], and *Gpr37l1<tm1d(EUCOMM)Wtsi>* strains was carried out. The results of the detailed comparative analysis were interpreted as confirming that the modulatory role of Gpr37l1 is specifically exerted in brain areas devoted to central cardiovascular control [45].

Other research focused on kidney-specific, somatic silencing of *Gpr37l1* expression by renal subcapsular infusion of small interfering RNA molecules in adult C57BL/6 wild-type mice, which resulted in increased urine output and sodium excretion, as well as decreased blood pressure [57].

## 4. Disease Modeling Applications

Autosomal recessive juvenile PD (Disease Ontology (DO) ID: 0060368, https://disease-ontology.org/?id=DOID:0060368, accessed on 20 March 2022; Online Mendelian Inheritance in Man (OMIM) ID: 600116, https://www.omim.org/entry/600116, accessed on 20 March 2022; Orphanet ID: 2828, https://www.orpha.net/consor/cgi-bin/OC_Exp.php?lng=EN&Expert=2828, accessed on 20 March 2022) is characterized by the early-onset, progressive and selective loss of *substantia nigra* dopaminergic neurons [9].

This distinctive trait has been modeled by both the *Prkn<tm1Ykt>* KO and the *Tg(Prp-GPR37)1Ryot* transgenic mouse strain, along with the *Prkn<tm1Ykt> Tg(PDGFB-GPR37)20Ryot* double mutant strain [19], in agreement with the previously observed, similar phenotype in *Prkn<tm1Ykt>* mice somatically expressing the human GPR37 protein, upon striatal injection of a recombinant adenoviral vector in *substantia nigra*’s neurons [20].

The expression of the *Tg(Prp-GPR37)1Ryot* transgene in *Prkn* wild-type mouse brain has also been reported to cause age-dependent, specific losses of dopaminergic neurons in striatum and other brain regions, in addition to increased susceptibility to the 6-OHDA dopaminergic neuron-specific toxin [18].

These findings are notably consistent with earlier studies of *Drosophila melanogaster* transgenic lines overexpressing the human GPR37 protein in dopaminergic neurons [58,59] and later analyses of rat (*Rattus norvegicus*) models overexpressing the endogenous GPR37 protein, following injection of a recombinant adeno-associated viral vector in the nigrostriatal system [60].

The independently established *Gpr37<tm2Gtva>* and *Gpr37<tm1.1Ryot>* mouse constitutive KO strains have both displayed variably decreased susceptibility to the above neurotoxins, diminished striatal dopamine levels, and mild locomotor hypoactivity [11,12,18].

The results of the above studies thus support the hypothesis that altered protein expression and/or neurotoxic events may lead to the intracellular mis-processing and aggregation of GPR37 receptor molecules, a condition which is strongly enhanced in the absence of efficient, parkin-mediated ubiquitination/proteasomal degradation, and constitutes a specific causative factor in the PD-associated, selective degeneration of *substantia nigra* neurons [11,12,18,19,20].

Different experimental outcomes have more recently been reported with *Gpr37<tm1Dgen>* null mutant mice, which present augmented sensitivity to the neurotoxic effects of 6-OHDA treatment and distinctive motor deficits at elder age [32].

More detailed comparative analyses may therefore be required to clearly distinguish the neurodegenerative effects of Gpr37 overexpression/intracellular retention, and the possible neuroprotective actions exerted by the receptor’s physiological expression at the neuronal plasma membrane. To this end, the establishment and phenotypic characterization of novel mouse mutant lines carrying nigrostriatal neuron- and age-specific KO alleles may be necessary, e.g., by crossing *Gpr37* conditional-ready, floxed mutant strains [11,18] to mutant mice expressing the cre recombinase protein in neuron-specific, developmental stage-inducible modalities.

Gpr37’s interaction with various membrane protein targets and consequent modulation of endoplasmic reticulum-associated protein degradation has also been studied by pull-down and co-precipitation assays in whole-brain samples from wild-type C57BL/6J mice and Wistar rats [61,62], as well as by *Gpr37* somatic expression silencing in ex vivo developing neurons from wild-type C57BL/6N mouse embryos [41]. Proteomics analysis of mouse and rat brain samples have also identified novel potential targets of Gpr37’s and Gpr37l1’s interaction [47,52,63].

More standardized, comparative investigations could then be conducted with mouse transgenic strains, allowing the cell type- and age-specific functional expression of wild-type Gpr37 and Gpr37l1 receptors and their possibly pathological variants, such as those present in familial cases of autism and seizure susceptibility [40,62].

Mammalian prosaposin proteins, derived saposin peptides, and synthetic prosaptide analogues exert cyto-trophic and -protective effects on a variety of cell types, at different developmental stages [64,65], and prosaposin deficiency causes a specific type of encephalopathy, a genetic lysosomal storage disease belonging to the group of sphingolipidoses [66] (https://disease-ontology.org/?id=DOID:0111330, accessed on 20 March 2022; https://www.omim.org/entry/611721, accessed on 20 March 2022; https://www.orpha.net/consor/cgi-bin/OC_Exp.php?lng=EN&Expert=139406, accessed on 20 March 2022).

Prosaposin and prosaptides have originally been proposed as Gpr37’s and Gpr37l1’s specific ligands, following in vitro cell expression and receptor signaling assays [4,6], as well as ex vivo somatic expression silencing by small interfering RNA molecules in cortical astrocytes from wild-type C57BL/6 mouse pups [5], or by AAV-micro RNA vectors in primary astrocytes or cortical neurons from brain samples of wild-type Wistar rat pups or embryos [6].

The prosaptide-induced, Gpr37- or Gpr37l1-mediated activation of intracellular signaling in certain mouse cell and tissue types has also been reported [16,49,56], as resulting from comparative ex vivo assays of wild-type and null mutant samples. Moreover, the concomitant expression of prosaposin and the two proposed receptors has been documented in various mouse tissues [16,17], as well as in glial and neuronal cells of developing or regenerating rat nerves [67,68].

However, other studies with *Gpr37* and *Gpr37l1* null mutant models of seizure susceptibility, ischemic stroke, and central cardiovascular control have not confirmed the co-expression of prosaposin and the two putative receptors, or the prosaptide-mediated activation of their intracellular signaling [31,40,44].

Thus, the establishment and phenotypic characterization of novel mutant models with variably combined cell type- and age-specific inducible ablation or expression of the *prosaposin*, *Gpr37*, and *Gpr37l1* genes may be required for the unequivocal assessment of the proposed ligand–receptor interaction, in both physiological and pathological conditions.

Indeed, the elucidation of Bglap/osteocalcin’s effects on oligodendrocyte differentiation and myelination via Gpr37 signaling has involved the complex, in vivo and ex vivo comparative analysis of constitutive *Bglap* KO and constitutive or tissue-specific *Gpr37* KO mouse models, along with the study of receptor function restoration in primary cell precursors from *Gpr37* null mutants, after transfection with Gpr37-expressing recombinant lentivirus vectors [39].

Similar approaches could then be applied to the characterization of the proposed interaction between neuroprotectin D1 and Gpr37 in macrophages and the resulting protective effects against infective sepsis and inflammatory pain [35,36].

Given the reported relation of human adenosine A2a receptor’s deleterious mutation and rare childhood-onset, infection-associated acute encephalopathies with epileptic seizures [69] (https://www.orpha.net/consor/cgi-bin/Disease_Search.php?lng=EN&data_id=22322, accessed on 20 March 2022), it would also be important to further extend the analysis of the presented interaction between the orthologous mouse adenosine A2a receptor and Gpr37 proteins in the hippocampus and striatum [23,24,25,26,27], as well as investigate the possible effects of Gpr37’s modulation on susceptibility to seizures [40].

Highly malignant, cerebellar MB [70] (https://disease-ontology.org/?id=DOID:0050902, accessed on 20 March 2022; https://www.omim.org/entry/155255, accessed on 20 March 2022) is among the most frequently diagnosed pediatric brain tumors. Shh-associated MB originates postnatally, from Shh-induced, dysregulated hyperproliferation of neuronal precursors in the developing cerebellum, as shown in heterozygous *Ptch1<tm1Zim>* KO and similar mouse models of human MB occurrence and progression [71,72].

Noticeably, *Gpr37l1* expression is altered in human MB [73], while the mouse Gpr37l1 protein has been shown to interact with Ptch1, the Shh-coreceptor, at the primary cilium membrane in developing cerebellar astrocytes. Gpr37l1 thus modulates Ptch1 intracellular trafficking, Shh production, and Shh-induced proliferation [8,48,49]. Moreover, double mutant mice carrying both the homozygous *Gpr37l1<tm1.2Gtva>* and the heterozygous *Ptch1<tm1Zim>* constitutive KO allele exhibit a marked delay in post-natal tumor occurrence and decreased incidence of more aggressive tumorigenic lesions [48].

More extensive studies of this double mutant model may then assist the detailed molecular and cytological characterization of the initial phases of Shh-associated MB occurrence and progression, as well as the pre-clinical assessment of potential applications to novel diagnostic and therapeutic approaches [48].

Comparable experimental strategies could also be considered for modeling, in vivo, Gpr37’s reported promotion of malignancy in some human tumor tissues and cell lines [74,75].

The functional analysis of the *Gpr37* and *Gpr37l1* genes can take great advantage of the current availability of co-isogenic C57BL/6N reporter-tagged, constitutive or cell type-specific KO mouse models. IKMC can in fact ensure the public of the ready provision of the suitable C57BL/6N-targeted ES cell clones and of an ample collection of isogenic C57BL/6N strains ubiquitously expressing the Flp or cre recombinase, or driving the cell type-specific expression of cre recombinase [53].

Such co-isogenic strains can constitute *ad hoc*, standardized genetic tools for the detailed, unbiased evaluation of complex phenotypic traits which have divergently been reported in previous publications, including, e.g., the proposed modulatory effects of Gpr37l1 on central cardiovascular control [45,55].

Co-isogenic C57BL/6N *Gpr37* and *Gpr37l1* mutant models can also be nominated and prioritized for standardized, high-throughput primary phenotyping by IMPC [76]. This leading global initiative systematically breeds at least one IKMC isogenic C57BL/6N KO strain, for each mouse gene [53]. Female and male mutant and sex-/age-matched wild-type control animals are then subjected to highly standardized in vivo and ex vivo phenotyping tests across a wide range of biological systems, at the embryonal stage, as well as early and late adult age. The resulting data are classified according to standard mammalian phenotype ontology definitions and made publicly and freely available to the international research community. Original programmatic comparisons are also carried out, allowing the automated, unbiased scoring of similar phenotypic traits in phenotype ontology-based descriptions of other mouse mutant models and human pathological conditions [76,77].

Notably, IMPC has ontologically classified so far observed phenotypic traits in published *Gpr37* and *Gpr37l1* mouse mutants and has processed them for comparative scoring, as described above. Several previously unreported associations between human diseases and each receptor’s gene have thus been predicted by significant phenotypic similarity, and may therefore constitute important insights for developing and pursuing novel lines of research (https://www.mousephenotype.org/data/genes/MGI:1313297#disease-section, accessed on 20 March 2022; https://www.mousephenotype.org/data/genes/MGI:1928503#disease-section, accessed on 20 March 2022).

## Figures and Tables

**Table 1:** Mouse *Gpr37* mutant strains.

Allele/Transgene Symbol (MGI ID) Type of Mutant/Transgenic Strain	Genetic Background	References
*Gpr37<tm2Gtva>* (MGI: 3027995) targeted constitutive KO strain obtained by crossing of *Gpr37<tm1Gtva>* (MGI: 3027992) targeted conditional-ready strain to *Tg(CMV-cre)1Cgn* (MGI: 2176180) cre recombinase-expressing strain	ES cells: 129P2/OlaHsd crossing: C57BL/6J N: 2–10 cre recomb.: BALB/c, C57BL/6	[11,12,13,14,15,16,17]
*Gpr37<tm1.1Ryot>* (MGI: 3768566) targeted constitutive KO strain obtained by in vitro cre recombinase expression in oocytes of *Gpr37<tm1Ryot>* (MGI: 3768565) targeted conditional-ready strain	ES cells: C57BL/6NSlc crossing: C57BL/6	[18]
*Tg(Prp-GPR37)1Ryot* (MGI: 3768632) brain-specific transgene-expressing strain	oocytes: C3H, C57BL/6 crossing: C57BL/6	[18,19]
*Tg(PDGFB-GPR37)20Ryot* (MGI: 3768633) midbrain, striatum-specific transgene-expressing strain	oocytes: C57BL/6 crossing: C57BL/6	[18,19]
*Prkn<tm1Ykt>* (MGI: 3698054), *Tg(Prp-GPR37)1Ryot* targeted constitutive KO, brain-specific transgene-expressing strain	*Prkn<tm1Ykt>*: ES cells: 129P2/OlaHsd crossing: C57BL/6 *Tg(Prp-GPR37)1Ryot*: as above	[19,20]
*Prkn<tm1Ykt>*, *Tg(PDGFB-GPR37)20Ryot* targeted constitutive KO, midbrain/striatum-specific transgene-expressing strain	*Prkn<tm1Ykt>*: as above *Tg(PDGFB-GPR37)20Ryot*: as above	[19,20]
*Gpr37<tm1Dgen>* (MGI: 3604579) targeted lacZ reporter-tagged constitutive KO strain	ES cells: 129P2/OlaHsd crossing: C57BL/6 N: 5–10	[17,21,22,23,24,25,26,27,28,29,30,31,32,33,34,35,36,37,38,39,40]
*Gpr37<em1Smoc>* (MGI: 6840940) oligodendrocyte-specific conditional KO line obtained by crossing of *Gpr37<em2Smoc>* (MGI: 6840941) CRISPR/Cas-mediated conditional-ready strain to an O*lig2*-cre recombinase transgene-expressing strain (not specified)	oocytes: C57BL/6J crossing: C57BL/6J cre recomb.: not specified	[39]

Abbreviations: MGI: Mouse Genome Informatics (http://www.informatics.jax.org/, accessed on 20 March 2022); KO: knock-out mutant; ES cells: background strain of targeted mutant embryonic stem (ES) cells; oocytes: background strain of transgene- or CRISPR/Cas-injected oocytes; crossing: background strain of wild-type mice used in crossing/backcrossing; N: number of backcross generations; cre recomb.: background strain of cre recombinase-expressing mice.

**Table 2:** Mouse *Gpr37l1* mutant strains and *Gpr37/Gpr37l1* double mutant strain.

Allele/Transgene Symbol (MGI ID) Mutation Type	Genetic Background	References
*Gpr37l1<tm1Mktk>* (MGI: 6763374) targeted constitutive KO strain	ES cells: 129S1/Sv crossing: C57BL/6	[43,44,45]
*Tg(Myh6-Gpr37l1)#Mktk* (MGI: 6763370) cardiac tissue-specific transgene-expressing strain	oocytes: not specified crossing: not specified	[43,45]
*Gpr37l1<tm1.2Gtva>* (MGI: 5439169) targeted constitutive KO strain obtained by crossing of *Gpr37l1<tm1.1Gtva>* (MGI: 439168) targeted conditional-ready strain to *Tg(CMV-cre)1Cgn* (MGI: 2176180) cre recombinase-expressing strain	ES cells: 129S6/SvEvTac crossing: C57BL/6J N: 2–10 cre recomb.: BALB/c, C57BL/6	[8,17,46,47,48,49]
*Gpr37l1<tm1.2Gtva>*, *Ptch1<tm1Zim>* (MGI: 1857935) double targeted constitutive KO strain	*Gpr37l1<tm1.2Gtva>*: as above *Ptch1<tm1Zim>*: ES cells: 129S2/SvPas crossing: C57BL/6 N: >10	[48]
*Gpr37l1<tm1Lex>* (MGI: 3528734) targeted lacZ reporter-tagged constitutive KO strain	ES cells: 129S/SvEvBrd crossing: C57BL/6 N: 2–10	[38,40,50,51]
*Gpr37l1<tm1Lex>*, *Gpr37<tm1Dgen>* double targeted lacZ reporter-tagged constitutive KO strain	*Gpr37l1<tm1Lex>*: as above *Gpr37<tm1Dgen>*: as above (Table 1)	[38,40,52]
*Gpr37l1<tm1b(EUCOMM)Wtsi>* targeted lacZ reporter-tagged constitutive KO strain obtained by crossing of *Gpr37l1<tm1a(EUCOMM)Wtsi>* (MGI: 4441736) targeted lacZ reporter-tagged conditional-ready strain to *Tg(CMV-cre)1Cgn* cre recombinase-expressing strain	ES cells: C57BL/6N-A<tm1Brd> crossing: C57BL/6 cre recomb.: BALB/c, C57BL/6	[45,53,54,55]
*Gpr37l1<tm1d(EUCOMM)Wtsi>* (MGI: 6150820) targeted reporter-free constitutive KO strain obtained by initial crossing of *Gpr37l1<tm1a(EUCOMM)Wtsi>* (see above) to *Tg(ACTFLPe)9205Dym* (MGI:2448985) Flp recombinase-expressing strain and crossing of resulting progeny to *Tg(CMV-cre)1Cgn* cre recombinase-expressing strain	ES cells: C57BL/6N-A<tm1Brd> crossing: C57BL/6 Flp recomb.: SJL, C57BL/6 cre recomb.: BALB/c, C57BL/6	[45,53,54,55]

Abbreviations: MGI: Mouse Genome Informatics (http://www.informatics.jax.org/, accessed on 20 March 2022); KO: knock-out mutant; ES cells: background strain of targeted mutant embryonic stem (ES) cells; oocytes: background strain of transgene-injected oocytes; crossing: background strain of wild-type mice used in crossing/backcrossing; N: number of backcross generations; cre recomb.: background strain of cre recombinase-expressing mice; Flp recomb.: background strain of flippase recombinase-expressing mice.

## Data Availability

Not applicable.
